# Fabrication and growth mechanism of t-selenium nanorods during laser ablation and fragmentation in organic liquids

**DOI:** 10.3389/fchem.2024.1449570

**Published:** 2024-09-20

**Authors:** Ilya V. Baimler, Alexander V. Simakin, Anastasia O. Dikovskaya, Valery V. Voronov, Oleg V. Uvarov, Alexander A. Smirnov, Alexey V. Sibirev, Alexey S. Dorokhov, Sergey V. Gudkov

**Affiliations:** ^1^ Prokhorov General Physics Institute of the Russian Academy of Sciences, Moscow, Russia; ^2^ Federal State Budgetary Scientific Institution “Federal Scientific Agroengineering Center VIM” (FSAC VIM), Moscow, Russia; ^3^ Institute of Biology and Biomedicine, Lobachevsky State University of Nizhni Novgorod, Nizhni Novgorod, Russia

**Keywords:** laser ablation, laser fragmentation, nanoparticles, selenium, selenium nanorods

## Abstract

**Introduction:**

The process of forming selenium nanoparticles with various shapes and structures through laser ablation and fragmentation in various solvents has been explored.

**Methods:**

Laser ablation and laser fragmentation techniques were employed using nanosecond Nd:YAG second harmonic laser irradiation in 9 different working fluids, including water. The characteristics of the resulting nanoparticles were assessed using transmission electron microscopy (TEM), dynamic light scattering (DLS), spectroscopy, and X-ray diffraction (XRD) methods.

**Results:**

Laser ablation and subsequent laser fragmentation of some organic solvents, such as ethanol, propanol-2, isobutanol, polyethylene glycol, and diethanolamine, have been found to produce trigonal selenium in the form of elongated nanorods approximately 1 μm long and 200 nm thick, with a well-defined crystal structure. In contrast, the use of deionized water, acetone, glycerol, and benzene as solvents results in the formation of spherical amorphous nanoparticles approximately 100 nm in diameter.

**Discussion:**

The polarity of the solvent molecules has been shown to influence the growth of crystalline selenium nanorods in solution during laser ablation and laser fragmentation. Generally, polar solvents hinder the growth of crystalline nanorods, due to interactions between selenium and solvent molecules. Nonpolar solvents, on the other hand, allow for laser fragmentation to reduce particle size and initiate the epitaxial growth of elongated, crystalline selenium nanorods.

## 1 Introduction

Recently, selenium nanoparticles have attracted increasing attention due to their excellent photovoltaic and semiconducting properties and high biological activity. Selenium is successfully used in solar cells (Hadar et al., 2019), rectifiers, and phototransistors (Qin et al., 2017). Selenium is also an essential trace element for all animals and plants (Turovsky et al., 2024). Selenium has been shown to prevent damage to cells and tissues from reactive oxygen species (Serov et al., 2023; Gudkov et al., 2020a). This fact justifies the use of selenium in agriculture, food industry and medicine (Baryshev et al., 2023).

A lot of research has been devoted to the production of selenium nanoparticles, and currently the methods used are divided into 3 groups: chemical, physical and biological methods (Chaudhary et al., 2016). Specific methods for obtaining selenium nanoparticles include the photocatalytic method, photothermal synthesis, biosynthesis, solid-state synthesis, vapor deposition technique, hydrothermal synthesis, electrodeposition, microwave synthesis, sonochemical synthesis, laser ablation technique in liquid (Skalickova et al., 2017). It should be noted that the laser ablation method on the one hand allows you to control the geometric parameters of nanoparticles, on the other hand it allows you to obtain chemically pure nanoobjects (Fromme et al., 2024; Ashikkalieva et al., 2023; Nastulyavichus et al., 2023; Cherepakhin et al., 2024; Ashikkalieva et al., 2022; Shabalina et al., 2022).

It is known that elemental selenium has several allotropic modifications: two amorphous forms (red and black) and three crystalline forms (dark red, gray and rhombic) (Ruiz‐Fresneda et al., 2023). Crystalline trigonal selenium (t-Se, also known as rhombohedral selenium) is of particular interest because it is a p-type semiconductor with an indirect bandgap of 1.6 eV and exhibits pronounced second harmonic generation and linear electro-optical effects (Cheng et al., 2019). Trigonal selenium, due to its high photoconductivity and high spectral sensitivity, has a wide range of applications in the production of solar cells, photometric lux meters, sensors and electrical rectifiers (Gawęda et al., 2009; Rasche et al., 2019). From a biological point of view, crystalline selenium, which has a needle-like structure, can disrupt the integrity of cells and lead to their damage, in contrast to amorphous selenium, which usually has a spherical shape (Varlamova et al., 2023). Therefore, the phase transition of selenium from an amorphous to a crystalline state is important from both biological and technological point of view.

As has been shown, the need for the synthesis of t-Se is due to the unique physical and chemical properties of this material. The production of selenium in the trigonal modification is possible using various techniques. The preparation of t-Se using vapor deposition (Suzuki et al., 2020), hydrothermal (An et al., 2003), sonochemical (Mayers et al., 2003), and chemical (Fan et al., 2005) methods has been reported.

The synthesis of selenium nanoparticles by laser ablation and fragmentation in aqueous solutions has been well studied and is used to create spherical nanoparticles of various sizes (Van Overschelde et al., 2013; Ayyyzhy et al., 2019). It is reported that using laser ablation of selenium powder, followed by deposition and heating at different temperatures, it is possible to control the thickness and length of t-Se crystals (Jiang et al., 2003). However, the production of trigonal selenium nanoparticles using laser ablation techniques in liquids has not been previously reported. In this work, we demonstrated for the first time the possibility of obtaining trigonal crystalline selenium using laser ablation and fragmentation methods in various solvents without the use of additional substances and additional effects on the colloid of nanoparticles.

## 2 Materials and methods

### 2.1 Preparation of spherical amorphous selenium nanoparticles using laser ablation

Spherical selenium nanoparticles were obtained using a laser ablation technique in a liquid. A polished target made of polycrystalline pure Se (99.99%) with dimensions of 2 × 2 cm was placed in a glass cell with a volume of 30 mL and placed at the bottom of the cell. The thickness of the liquid layer between the surface of the target and the liquid was 2-3 mm. Deionized water (18 MΩ × cm) was used as working liquid. During the ablation process, a volume of liquid was constantly pumped inside the cuvette at a rate of 0.5 mL/s using a peristaltic pump connected to an additional reservoir. The total volume of the system was approximately 70 mL. An Nd:YAG laser NL300 (Ekspla, Vilnius, Lithuania) with the following parameters was used as a source of laser radiation: pulse duration τ = 4 ns, frequency υ = 1 kHz, wavelength λ = 532 nm, pulse energy ε = 2 mJ. During irradiation, the beam was moved along the target surface using a galvanomechanical scanner LScanH (Ateko-TM, Moscow, Russia) and an F-Theta lens with a focal length of 90 mm. The spot size in the beam waist was 100 µm. The energy density of laser radiation on the target surface was 25 J/cm^2^. The beam trajectory consisted of several parallel lines inscribed in a 1 × 1 cm square. The scanning speed was 3000 mm/s. Typical ablation time was approximately 30 min.

### 2.2 Laser fragmentation of selenium nanoparticles

Se nanoparticles obtained by ablation in water were transferred to isopropanol by particle precipitation by centrifugation and subsequent solvent replacement. Initially, Se nanoparticles were precipitated using a LMC-4200 centrifuge (Biosan, Riga, Latvia). Centrifugation was performed at 15,000 rpm for 40 min. Deionized water was withdrawn from the colloid with precipitated nanoparticles and chemically pure propanol-2 was poured instead of water. The resulting colloid was then placed in an ultrasonic bath (ultrasonic power was 20 W) for 2-3 min. The described procedure of particle precipitation and replacement of the solvent with pure propanol-2 was performed 4 times. Selenium particles after ablation in water were transferred to other solvents by a similar method.

The resulting colloidal solution of amorphous selenium nanoparticles were subjected to further laser fragmentation. During laser fragmentation, colloidal solution of selenium nanoparticles obtained as result of laser ablation was placed in glass cuvette with a transparent bottom, and the radiation was focused using an F-Theta lens at a distance of 1 cm from the bottom of the cuvette. The radiation entered the cell from below in order to avoid radiation scattering by gas bubbles, which were intensively formed in the cell as result of breakdown. Typical laser fragmentation time was approximately 60 min.

### 2.3 Characterization of selenium nanoparticles

A DC24000 analytical centrifuge (CPS Instruments, Oosterhout, the Netherlands) and a Zetasizer Ultra RedLebel 10 dynamic light scattering setup (Malvern Panalytical, Malvern, United Kingdom) were used to study the size distribution of nanoparticles and determine the concentration of nanoparticles (Gudkov et al., 2020b). The extinction spectrum of colloidal solutions of selenium nanoparticles was studied using a USB3000T spectrometer (Ocean Optics) (200–800 nm) and a two-channel spectrometer UV-3600 Series (Shimadzu) (300–1,600 nm). The spectra were measured in quartz cuvettes with volume of 3 mL, the optical path length was 1 cm (Sevostyanov et al., 2020). The extinction spectra of the corresponding working fluids used in laser ablation and fragmentation were used as reference spectra. The crystal structure of selenium nanoparticles was studied using a Bruker AXS P4 X-ray diffractometer (Bruker, Billerica, MA, United States). A Libra 200 FE HR transmission electron microscope (Carl Zeiss, Jena, Germany) was used to obtain TEM images of particles and study their morphology. When preparing nanoparticles for TEM microscopy, gold microscopic grids were used.

### 2.4 Effect of ultrasound on colloidal solutions of nanoparticles

Colloidal solutions of selenium nanoparticles obtained by ablation were placed in glass containers with volume of 10 mL. Then these containers with the colloid of nanoparticles were placed in an ultrasonic bath VBS-3D (Vilitek, Russia) with an ultrasound power of 120 W, the time of exposure to ultrasound on the colloid was regulated using a built-in timer at intervals of 10, 30, 50, 70, 90, and 110 min, the temperature during ultrasound exposure was maintained at 25°C. The solutions in the ultrasonic bath were positioned so that the water level in the bath was approximately 2/3 of the height of the glass vessel with the colloidal solution.

### 2.5 Effect of temperature on colloidal solutions of nanoparticles

A drop of an aqueous colloidal solution in volume of 5 μL was placed on a hard polished and heat-treated silicon wafer measuring 1 × 1 cm using an automatic dispenser. After the drop dried on the silicon surface, the initial images of the nanoparticles were recorded using an optical microscope. Then the sample was exposed to temperature in an electric furnace with a TP703 thermostat (NPK Varta, St. Petersburg, Russia). In the experiments, the parameters of temperature and heating time of a silicon wafer with selenium nanoparticles were adjusted. Images of selenium nanostructures and nanoparticles on silicon substrate were obtained using an MXR optical metallographic microscope (Erstevak ltd.).

### 2.6 Statistics

Mean and standard error of the mean (SEM) were calculated for 5 measurements over at least 3 samples for most variables.

## 3 Results

### 3.1 Morphology of obtained selenium nanoparticles

**FIGURE 1:**
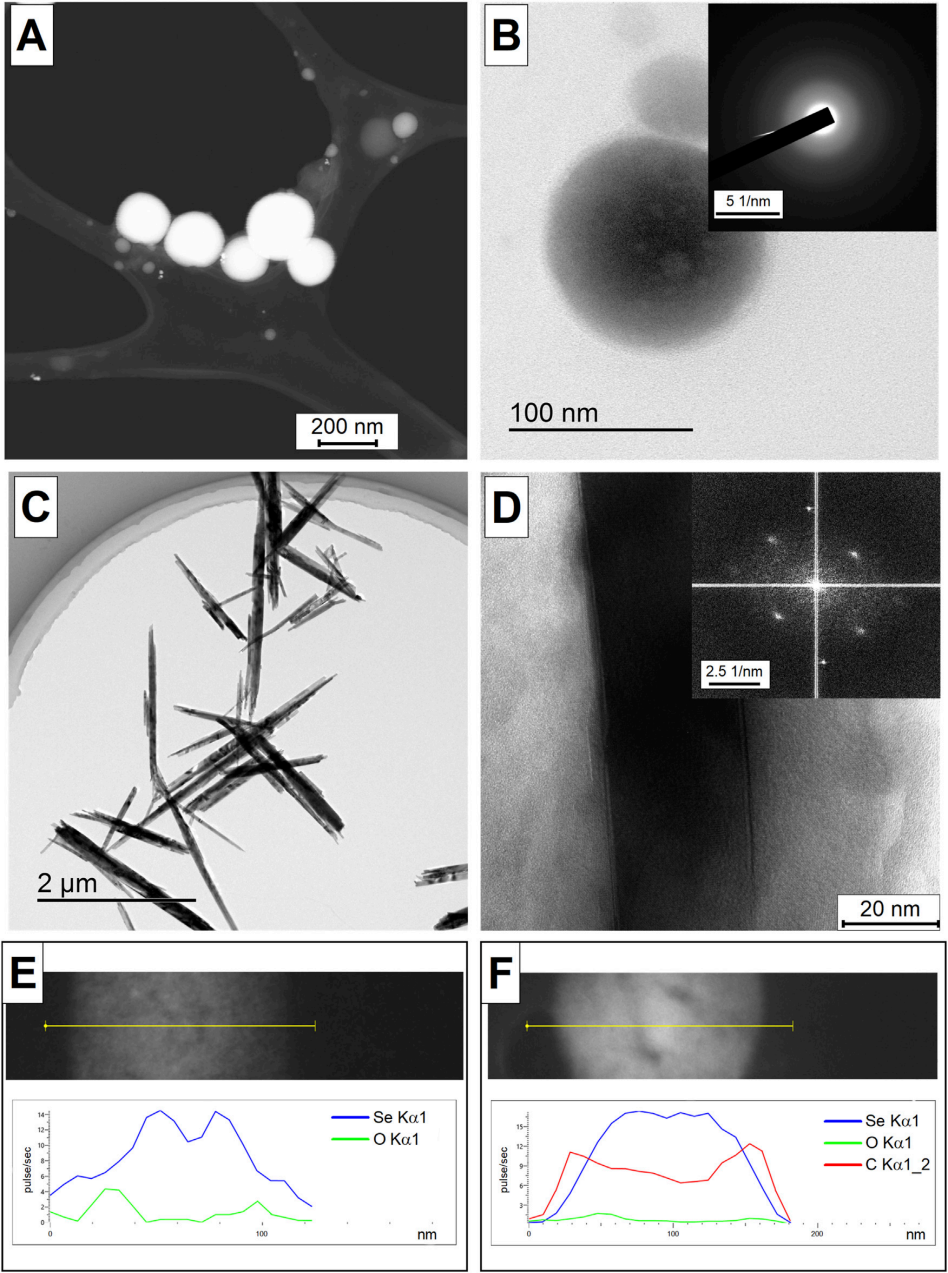
TEM images of selenium nanoparticles obtained using laser ablation technique in water **(A)**. HRTEM image of an individual selenium nanoparticle obtained in aqueous solution as result of laser ablation **(B)** and the corresponding diffraction pattern in the inset in the upper right corner. TEM images of selenium nanoparticles obtained using the laser fragmentation technique in isopropanol **(C)**. HRTEM image of an individual selenium nanoparticle obtained as result of laser fragmentation in isopropanol **(D)** and the corresponding diffraction pattern in the inset in the upper right corner. EDX-obtained distribution of Se and O atoms on Se particle obtained after laser ablation in water **(E)**. EDX-obtained distribution of Se, O and C atoms on Se particle obtained after 10 min of laser fragmentation in propanol-2 **(F)**. Analysis of the EDX element distribution obtained along the yellow line in the corresponding TEM images.

TEM images of selenium nanoparticles obtained as result of laser ablation and subsequent laser fragmentation in water (Figures 1A, B) and isopropanol (Figures 1C, D) are presented. It has been shown that selenium nanoparticles obtained in water have spherical shape, the sizes of nanoparticles are in the range of 200 nm (Figure 1A). The diffraction pattern of an individual selenium nanoparticles presented in Figure 1B demonstrates the absence of pronounced diffraction reflections, which indicates that the selenium nanoparticles obtained as result of ablation are amorphous. Nanoparticles obtained by laser ablation and fragmentation in isopropanol have an elongated needle-like shape, the particle thickness is approximately 200 nm, and the length varies from 2 to 4 μm (Figure 1C). Figure 1D shows a magnified image of an individual selenium nanoparticle and its corresponding diffraction pattern. It was shown that the resulting elongated selenium nanoparticles have clearly defined crystalline structure with reflections corresponding to the (100), (101) and (110) directions. The distance between the atomic planet of the crystal for the indicated directions is about 3.8, 3, and 2.1 Å. The results of the energy dispersive X-ray spectroscopy (EDX) analysis of selenium nanoparticles obtained after laser ablation suggest the presence of oxygen in their composition (Figure 1E). However, upon laser fragmentation of these nanoparticles in propan-2 for a period of 10 min, the presence of both carbon and oxygen atoms was detected, in addition to selenium (Figure 1F). This is likely due to the formation of a carbon shell around the particles, a phenomenon commonly observed during laser ablation and fragmentation in organic solvents (Zhang et al., 2019).

**FIGURE 2:**
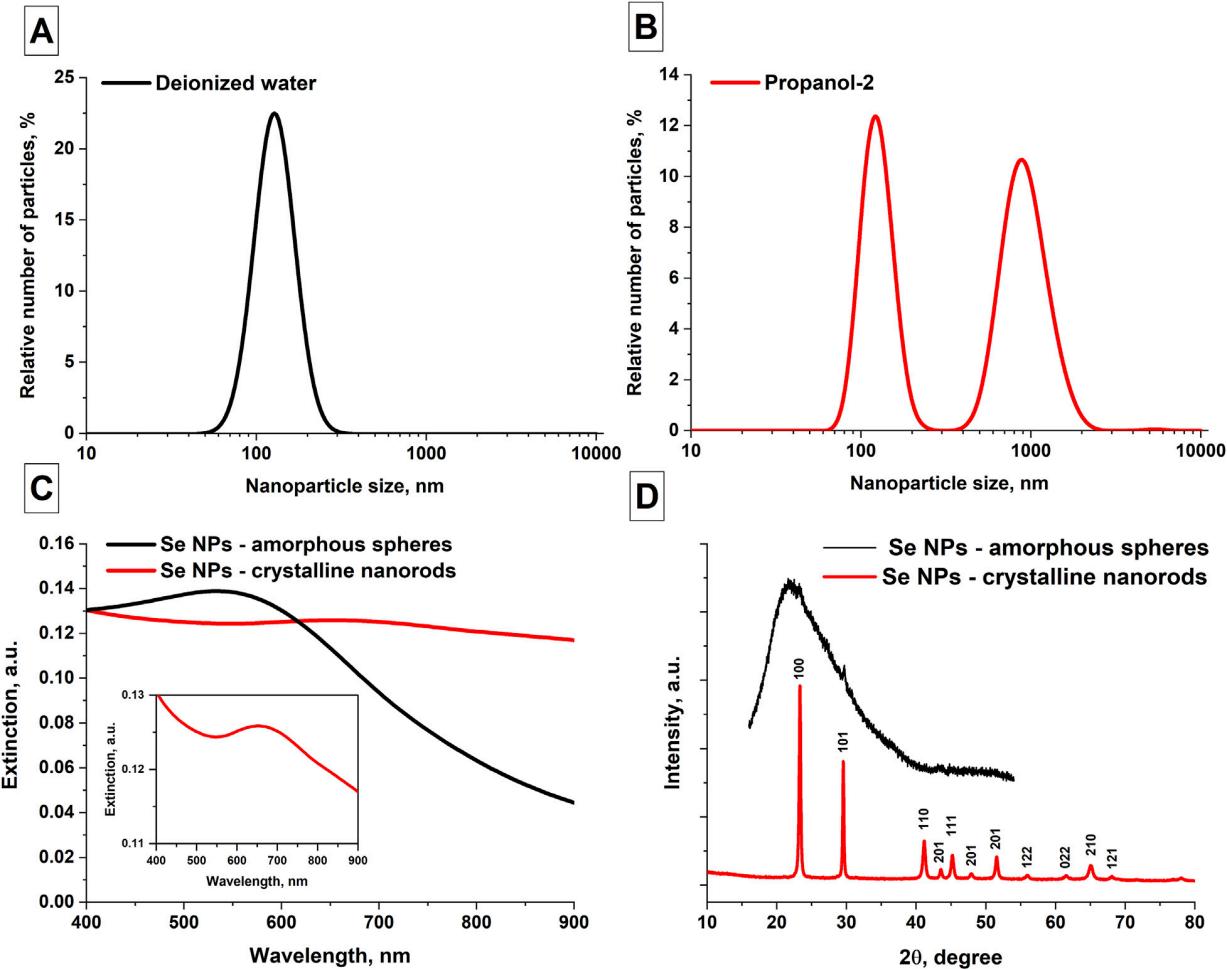
Spectral, phase and size characteristics of the obtained selenium nanoparticles. **(A)** Distribution of the number of Se NPs as function of size obtained in aqueous solution; data averaged over 5 measurements for 3 samples. **(B)** Distribution of Se NPs obtained by ablation and subsequent fragmentation in isopropanol; data averaged over 5 measurements for 3 samples. **(C)** Extinction spectrum of colloidal solutions of selenium nanoparticles obtained after ablation in water (black curve) and fragmentation in isopropanol (red curve), Inset figure: extinction spectrum of SeNRs. **(D)** X-ray diffraction pattern of selenium nanoparticles obtained by laser fragmentation in isopropanol. The inset in the upper right corner shows an X-ray diffraction pattern of selenium nanoparticles obtained by laser ablation in isopropanol.

Using DLS and disk centrifuge, the distribution of selenium nanoparticles obtained by laser ablation in various solvents was studied. It was shown that colloidal solutions of selenium nanoparticles obtained in deionized water have monomodal distributions (Figure 2A). The maximum distribution of particles in an aqueous colloid occurs at size of approximately 130 nm; the half-width of the distribution is approximately 40 nm. The obtained distributions of selenium nanoparticles synthesized in isopropanol show that there are several maxima in the distribution, i.e., the particle distribution is bimodal (Figure 2B). The maximum distribution of the number of selenium particles depending on size when using isopropanol as working fluid occurs at 120 nm and 900 nm, with corresponding half-widths of the distributions of approximately 25–30 nm and 300 nm.

The extinction spectra of colloidal solutions of selenium nanoparticles obtained using laser ablation and laser fragmentation techniques in various working fluids were studied. It has been shown that the main distinctive feature characteristic of the extinction spectrum of elongated selenium nanoparticles is the increase in extinction in the red region of the spectrum (Figure 2C). In the wavelength range of about 900 nm, the extinction of radiation by spherical nanoparticles is significantly less. In addition, for spherical nanoparticles the extinction maximum occurs at approximately 550 nm and the extinction spectrum of Se NR exhibits a maximum at a wavelength of 650 nm (Inset Figure 2C). Figure 2D shows X-ray diffraction patterns of two samples of selenium nanoparticles - an XRD image characteristic of nanoparticles obtained in isopropanol, as well as an X-ray diffraction pattern characteristic of nanoparticles obtained in water. The X-ray pattern obtained for selenium particles in propanol-2 corresponds to the trigonal phase of selenium. No other crystalline impurities were detected by XRD. The X-ray diffraction pattern of selenium particles obtained in water corresponds to the amorphous phase of nanoparticles, with an insignificant content of crystalline monoclinic and hexagonal phases.

### 3.2 Effect of ultrasound and temperature on Se nanoparticles obtained in water

**FIGURE 3:**
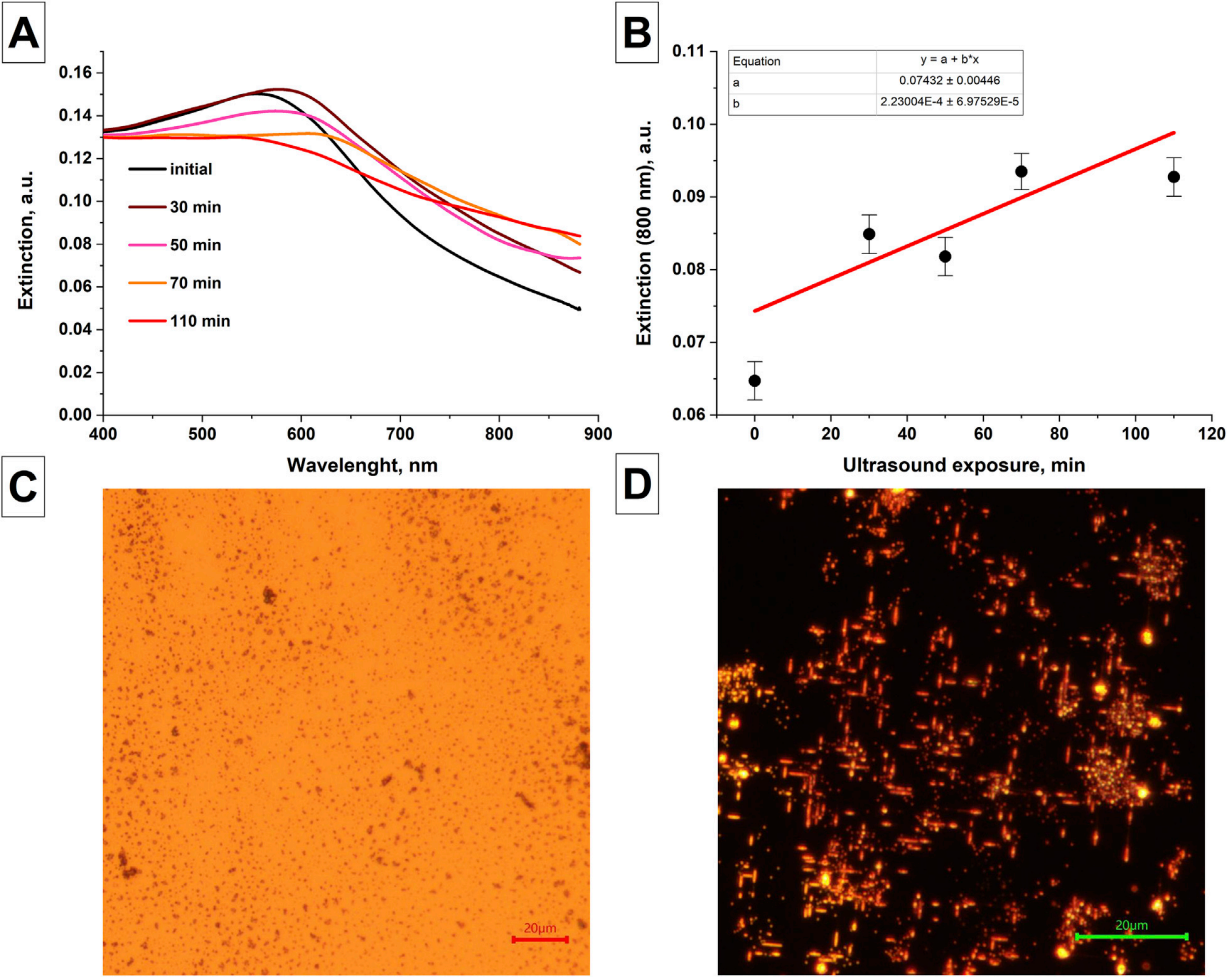
Changes in the characteristics of nanoparticles after exposure to ultrasound and temperature. **(A)** Changes in the extinction spectra of colloidal solution of selenium nanoparticles obtained after laser ablation depending on the duration of exposure of the colloid to ultrasound; Data averaged for 3 samples. **(B)** The magnitude of extinction of radiation at wavelength of 800 nm by colloidal solutions of selenium nanoparticles depending on the duration of ultrasonic exposure of the colloid. Images of selenium nanoparticles obtained using laser ablation in water under the influence of temperature. **(C)** Initial sample of selenium nanoparticles. The size of the scale mark in the image is 20 µm. **(D)** Reflected light images of selenium nanoparticles after temperature exposure T = 400°C, t = 30 min; The size of the scale mark in the image is 20 µm.

The change in the extinction spectra of colloidal solutions of selenium nanoparticles in isopropanol, obtained after laser ablation with prolonged exposure to ultrasound on the colloidal solution, was studied, Figure 3A. It was shown that the extinction spectrum of colloidal solutions of selenium nanoparticles under ultrasonic influence on the colloid shows an increase in the extinction in the range from 800 to 900 nm (Figure 3B). At the same time, the extinction peak characteristic of amorphous selenium at 550 nm begins to decrease with increasing time of ultrasonic exposure. As was shown earlier in Figure 2C, the trigonal modification of selenium is characterized by an increase in extinction in the red region of the spectrum and the disappearance of extinction peak at 550 nm, which is associated with change in the shape of nanoparticles.

It has been shown that with long-term (30 min) exposure to temperature of 400°C on silicon substrate coated with spherical selenium nanoparticles obtained by laser ablation in water, the formation of elongated selenium particles on the substrate is observed, Figures 3C, D.

In addition to water and isopropanol, other organic solvents have also been used as working liquids in laser ablation and fragmentation processes. At least three samples of selenium nanoparticles were obtained for each solvent. Table 1 provides information on the structure and shape of nanoparticles obtained by laser ablation for 30 min and fragmentation for 1 h using an appropriate solvent as well as the data on solvent dipole moments in liquid state.

**TABLE 1:** Effect of the liquid used in laser ablation and fragmentation on the properties of the resulting selenium nanoparticles.

Solvent type	Solvent molecule dipole moment µ, D	Shape and phase of selenium nanoparticles
Benzene (C_6_H_6_)	0	Spherical, amorphous
Polyethylene glycol (C_3n_H_6n+2_O_n+1_)	2.38 (Uchida et al., 1956)	Elongated, crystalline
Ethanol (C_2_H_5_O)	2.56 (Jorge et al., 2022)	Elongated, crystalline
Isobutanol (С_4_H_10_O)	2.69 (Rizk and Elanwar, 1968)	Elongated, crystalline
Propanol-2 (C_3_H_8_O)	2.71 (Jorge et al., 2022)	Elongated, crystalline
Diethanolamine (C_4_H_11_NO_2_)	2.78 (Hsieh et al., 2007)	Elongated, crystalline
Glycerol (C_3_H_8_O_3_)	2.81 (Rizk and Elanwar, 1968)	Spherical, amorphous
Deionized water (H_2_O)	2.95 (Gubskaya and Kusalik, 2002)	Spherical, amorphous
Acetone (C_3_H_6_O)	3.68 (Pereyra et al., 2011)	Spherical, amorphous

## 4 Discussion

In experiments on laser ablation of solid selenium target and subsequent fragmentation of colloidal solution of nanoparticles, it was shown that in the process of laser irradiation of colloidal solutions using deionized water, acetone, glycerol and benzene as solvents, spherical amorphous selenium nanoparticles are formed. When ethanol, propanol-2, isobutanol, diethanolamine, and polyethylene glycol are used as solvents, the formation of elongated selenium nanorods is observed. Thus, it is shown that, other things being equal, the transition of amorphous selenium from the amorphous phase to the trigonal phase and the further growth of selenium nanocrystals is associated with the solvent used in which the synthesis and irradiation of nanoparticles occurs.

**FIGURE 4:**
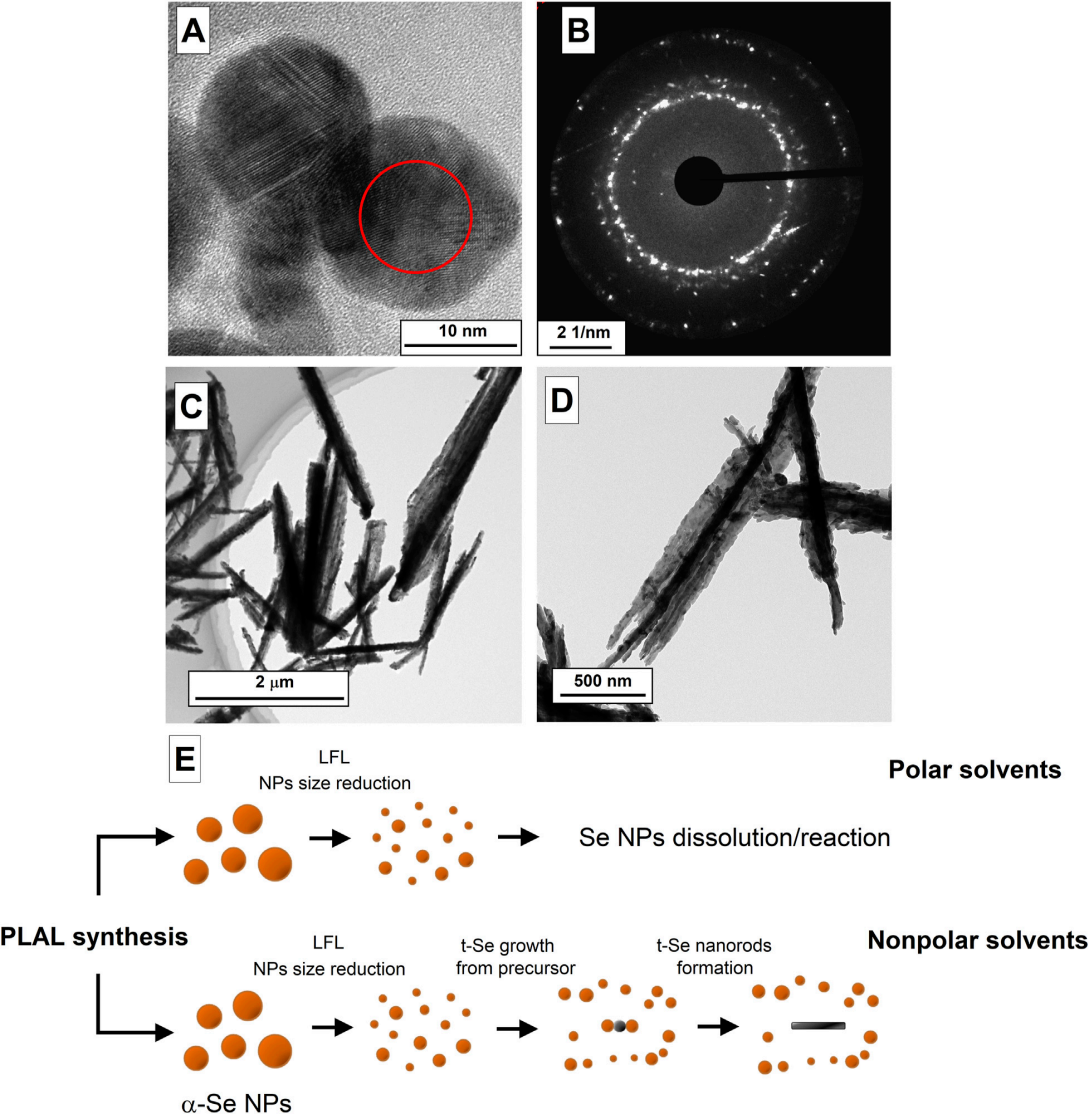
TEM images of selenium nanoparticles obtained after laser ablation in isopropanol **(A)** and the diffraction pattern corresponding to the TEM image **(B)**. **(C,D)** TEM images of t-Se nanoparticles obtained after laser fragmentation of selenium in mixture of ethanol and water. **(E)** Schematic representation of the formation process of amorphous and trigonal selenium by laser fragmentation.

During laser ablation and laser fragmentation of nanoparticle colloids, as result of laser-induced breakdown, the propagation of intense ultrasonic vibrations in the irradiated volume is observed, highly reactive molecular decomposition products are formed, and local heating of the colloid occurs to high temperatures. It is possible that as a result of the processes observed during laser ablation and laser fragmentation, some selenium particles in the colloid undergo transition from the initial amorphous state to the trigonal phase. Previously, in (Zhang et al., 2004; Yang et al., 2023), it was shown that exposure of solutions of selenium nanoparticles to ultrasonic vibrations leads to crystallization and further growth of elongated selenium particles. This is confirmed by our experiments with amorphous selenium particles, which were separately exposed to ultrasonic vibrations and temperature (Figures 4A, B). In colloid, such particles can play the role of precursor seeds, on which epitaxial growth of selenium crystal subsequently occurs.

Figure 4A shows TEM images of selenium nanoparticles that are present in the colloid after the stage of laser ablation and fragmentation during 10 min in isopropyl alcohol. It can be seen that particles about 15 nm in diameter have the spherical shape (Figure 4A), as in the case of ablation in water and acetone, but the images clearly show the crystal lattice, which is also confirmed by the diffraction pattern, in which individual reflections are visible (Figure 4B). Thus, the transition of amorphous selenium nanoparticles to trigonal crystalline modification under the action of laser-induced breakdown of colloids is confirmed.

The article (Chen et al., 2010) describes a method for producing trigonal modification particles in aqueous solutions of Na_2_SeO_3_ and glucose and the growth of selenium crystals in aqueous solutions and ethanol. It is noted that the growth rate of selenium crystals is different for aqueous solutions and ethanol, since the dissolution of amorphous selenium in ethanol occurs faster than in water. It is also noted that the growth of crystalline selenium in aqueous solutions can be caused by the presence of glucose in the solution, which can play the role of surfactant that regulates the growth of selenium crystals. The role of surfactants in the formation of trigonal selenium crystals is noted in other works, for example, in (Ma et al., 2004; Ma et al., 2015). In our work, the growth of crystalline selenium in the trigonal modification does not occur in some solvents, even though the solution was subjected to intense thermal and ultrasonic exposure as a result of optical breakdown, which may indicate the key role of surfactants in the growth process of t-Se crystals and the influence oxidative processes.

The growth of crystalline selenium in an aqueous solution is most likely limited by the oxidation process and the appearance of selenium dioxide SeO_2_ in the solution, which is readily soluble in water at room temperatures: Se + 2H_2_O → SeO_2_ + 2H_2_. During laser fragmentation, the particle size gradually decreases with increasing irradiation time. In this case, the minimum size of selenium particles turns out to be limited from below, since small particles turn out to be completely oxidized, while submicron selenium particles represent core-shell structures Se@SeO_2_, the presence of which is confirmed by EDX spectroscopy (Figure 1E). Therefore, in colloid of nanoparticles after laser irradiation, selenium is found in the form of spherical amorphous particles with sizes ranging from several tens of nanometers to hundreds of nanometers and dissolved SeO_2_. In the case of organic solvents (ethanol, propanol-2, isobutanol, etc.) in the colloid it is possible to obtain fairly small fraction of nanoparticles (a few nanometers), which are precursors for the growth of trigonal selenium crystals. To confirm this hypothesis, an experiment was conducted with mixture of ethanol and deionized water (80%–20%). As a result of laser fragmentation, the presence of deformed selenium crystals was observed in the solution (Figures 4C, D); the destruction of selenium crystals occurs due to the oxidation of selenium by oxygen present in water, which then dissolves in ethanol and water. Apparently, selenium behaves in similar way in polar solvents (Steichen and Dale, 2011; Langi et al., 2010), where selenium particles interact with solvent molecules, for example, as follows: 2Se + 2C_3_H_6_O → 2C_3_H_5_OSe + H_2_. It is reported that using various nonpolar solvents during the synthesis of trigonal selenium, it is possible to obtain crystalline 2D nanostructures in the form of ribbons of crystalline selenium (Ma et al., 2015).

A schematic representation of the selenium nanorod formation process during fragmentation is shown in Figure 4E. As a result of ablation of the solid target of selenium nanoparticles, large nanoparticles (100–200 nm) of amorphous α-Se are present in the colloid. As a result of laser fragmentation, the size of nanoparticles decreases; Along with this, the concentration of nanoparticles increases, and t-Se precursor particles are formed under the influence of high temperatures and ultrasound. Next, small fraction of amorphous selenium nanoparticles, depending on the type of liquid selected (polar or non-polar), can dissolve in colloid (polar liquids), thereby leaving only fraction of particles with large sizes, or will interact with t-Se precursor particles providing epitoxic growth of selenium nanorods.

It’s interesting to note that laser fragmentation of selenium nanoparticles in benzene leads to the formation of spherical particles rather than nanorods as originally thought, since the benzene molecule is nonpolar. This fact may indicate that selenium in the form of nanoparticles in colloid can react with benzene molecules under the action of external factors (laser radiation, plasma, thermal heating, ultrasound) to form compounds such as selenophenes, selenobenzene and others. However, this assumption requires further studies.

Thus, this work demonstrates for the first time, method for producing trigonal modification of selenium in the form of elongated nanorods using laser ablation and fragmentation in the liquids without the use of additional precursors and surfactants. It has been shown that the polarity of the solvent molecules influences the growth of crystalline selenium nanorods in solution during laser ablation and laser fragmentation. In polar solvents, the growth of crystalline selenium nanorods is inhibited by the oxidation of selenium or its interaction with solvent molecules. One of the possible mechanisms for the formation of crystalline selenium nanorods may be the growth of crystals from t-Se precursor particles formed under the influence of temperature and ultrasound. In non-polar solvents, laser fragmentation reduces particle size, initiating epitaxial crystal growth, while in polar solvents, small selenium particles interact with solvent molecules and thereby limit nanocrystal growth.

## Data Availability

The raw data supporting the conclusions of this article will be made available by the authors, without undue reservation.
